# How is ‘shortage’ defined? Exploring Nursing Workforce Data across Canada 2015-2022: An Ecological Study

**DOI:** 10.23889/ijpds.v9i5.2733

**Published:** 2024-09-18

**Authors:** Megan Harmon, Riley Martens, Shabnam Vatanpour, Natalie Sapiro, Robin Walker, Tracie Risling, Cathy Eastwood

**Affiliations:** 1 University of Calgary

## Objective

Determining the ability of a country’s nursing workforce to meet the health care needs of the population is essential for optimal health outcomes. ‘Nursing shortage’ is frequently heralded as an issue, yet it is unclear how ‘shortage’ is defined and calculated. The purpose of this study was to collect and link publicly available Canadian data to describe and compare trends in nursing workforce capacity.

## Methods

Primary data sources included linking Statistics Canada and Canadian Institutes of Health Information (CIHI) data from 2015 to 2022. Statistics Canada tracks provincial population data and job vacancy rates. CIHI receives data from provincial nursing organizations on demographics, roles, and employment status. To estimate a sufficient workforce, job vacancy rates (a proxy for provincial need) were cross tabulated with the number of registered nurses (RNs) and registered psychiatric nurses (RPNs) per year.

## Results

The number of RNs and RPNs in Canada has increased by 8.6% between 2015 and 2022, to a total of 322,226. Job vacancies, as a percent of total nursing supply, shows a rising trend (2.3% to 8.7%) between 2015 and 2022 across Canada. In 2022, 84.9% of Canadian RNs and RPNs in direct patient care across Canada and 86.1% were in urban settings.

## Conclusion & Limitations

This project examines Canadian nursing workforce data encompassing potential effects of the COVID-19 pandemic. The trends are limited to annual due to aggregated data. Data on a country’s nursing workforce measured monthly and consistently across provinces would yield clearer information.

